# Successful CT-Guided Thrombin Injection of a Peripancreatic Left Gastric Artery Pseudoaneurysm in Chronic Pancreatitis

**DOI:** 10.7759/cureus.111187

**Published:** 2026-06-20

**Authors:** Genevieve Crotty, Wasim Awal

**Affiliations:** 1 Anesthesiology and Critical Care, Prince of Wales Hospital, Sydney, AUS; 2 Radiology, Gold Coast University Hospital, Gold Coast, AUS

**Keywords:** complications of pancreatitis, ct-guided thrombin injection, endovascular aneurysm repair, percutaneous procedure, pseudoaneurysm

## Abstract

Visceral artery pseudoaneurysms are rare but serious complications of pancreatitis that can be challenging to manage when conventional endovascular approaches are not feasible. We describe the case of a 65-year-old man with chronic pancreatitis who developed a small visceral pseudoaneurysm that could not be safely accessed for embolization because of challenging vascular anatomy. A minimally invasive CT-guided percutaneous thrombin injection was performed, resulting in successful thrombosis and resolution on follow-up imaging. This case highlights an alternative treatment strategy in technically difficult scenarios and underscores the importance of imaging in both diagnosis and postprocedural assessment.

## Introduction

Visceral artery pseudoaneurysms are uncommon but potentially life-threatening complications of pancreatitis, occurring in both acute and chronic settings. Pancreatic inflammation can lead to autodigestion of adjacent arterial walls by proteolytic enzymes, resulting in vessel wall necrosis, pseudoaneurysm formation, and, if untreated, catastrophic hemorrhage [1,2]. Pseudoaneurysms most frequently involve arteries in close proximity to the pancreas, such as the splenic, gastroduodenal, pancreaticoduodenal, and left gastric arteries [3,4].

Although rare, with an estimated incidence of 1-10% in patients with pancreatitis, pseudoaneurysms carry substantial morbidity and mortality because of their propensity to rupture [5]. Unlike true aneurysms, pseudoaneurysms lack all three layers of the vessel wall and are instead contained by surrounding fibrous tissue, making them inherently unstable. Their clinical presentation can vary from vague abdominal pain to overt GI bleeding or hemodynamic collapse, depending on the site and severity of rupture [5,6].

Diagnosis is typically achieved through contrast-enhanced CT, which not only identifies the pseudoaneurysm but also assesses the severity of the underlying pancreatitis. Arterial-phase CT provides superior sensitivity compared with ultrasound or portal venous-phase CT and is essential for mapping vascular anatomy and planning subsequent angiography [5,6]. In most centers, CT findings prompt digital subtraction angiography (DSA), which remains the gold standard for both definitive diagnosis and treatment through selective catheterization and embolization of the feeding artery [2,3]. However, technical challenges such as vessel tortuosity, small caliber, or inaccessibility may preclude successful endovascular treatment.

In this setting, percutaneous image-guided thrombin injection has emerged as an alternative minimally invasive technique. Initially developed for iatrogenic femoral artery pseudoaneurysms, its use has since been extended to selected visceral artery pseudoaneurysms when conventional endovascular approaches are not feasible [7,8]. When performed under CT or ultrasound guidance, thrombin induces rapid intraluminal thrombosis of the pseudoaneurysm sac, offering a potentially safe and effective therapeutic option in appropriately selected patients [4-9].

In this report, CT-guided percutaneous thrombin injection is presented as a minimally invasive alternative for visceral artery pseudoaneurysms when standard endovascular embolization is not feasible because of unfavorable anatomy.

## Case presentation

A 65-year-old man with a history of chronic pancreatitis presented to the emergency department with a two-week history of worsening intermittent epigastric pain and episodes of melena. He reported longstanding epigastric discomfort and had previously been diagnosed with gallstone pancreatitis in 2021, for which he underwent a laparoscopic cholecystectomy. This procedure initially provided symptomatic relief; however, his symptoms gradually recurred over the ensuing years.

Three years after laparoscopic cholecystectomy, he re-presented with recurrent, progressively worsening epigastric pain. On examination, his abdomen was diffusely tender with voluntary guarding; however, he remained hemodynamically stable. Initial blood tests were largely unremarkable, stable, and nondiagnostic, with hemoglobin 120 g/L, lactate 2.4 mmol/L, lipase 68 U/L, and normal liver function tests. In the context of ongoing diagnostic uncertainty, he was admitted under the surgical team for further evaluation.

Over the subsequent days, he remained hemodynamically stable but continued to experience persistent abdominal pain. Liver function tests became transiently deranged, demonstrating a mixed hepatocellular and cholestatic pattern, and inflammatory markers rose (peak C-reactive protein, 132 mg/L), whereas hemoglobin remained above 115 g/L. In light of these evolving biochemical abnormalities and persistent symptoms, cross-sectional imaging was pursued. Arterial-phase contrast-enhanced CT demonstrated features of acute pancreatitis with a probable pancreatic pseudocyst and a suspected visceral pseudoaneurysm (Figure 1). Given these findings, he was transferred to a tertiary center for DSA and consideration of endovascular embolization.

**Figure 1:**
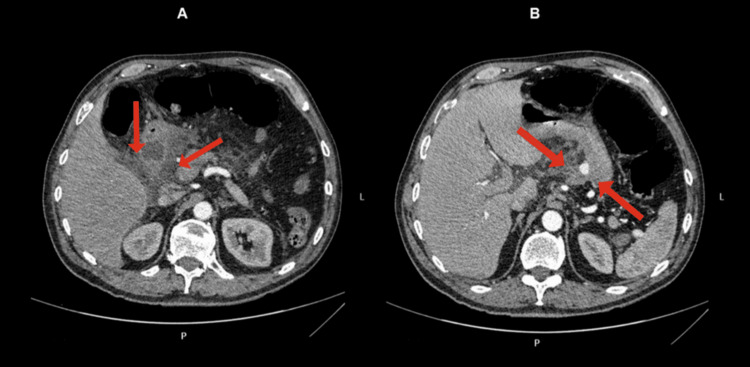
Arterial-phase contrast-enhanced CT of the upper abdomen demonstrating acute pancreatitis with peripancreatic collections (A) and a contrast-opacified pseudoaneurysm superior to the pancreatic body (B), arising in close proximity to the left gastric artery

After transfer, DSA was performed to confirm the diagnosis and facilitate potential therapeutic embolization. The angiogram identified an 8-mm pseudoaneurysm arising via a 2-mm neck from an inferior branch of the left gastric artery. A Cobra catheter was advanced into the left gastric artery; however, despite microcatheter placement at the branch origin, the operators were unable to navigate into the horizontal segment to permit coil or other embolic deployment. Fortunately, at the time of DSA, hemoglobin levels and vital signs remained stable, allowing further consideration of management options.

In view of the failed endovascular approach due to complex vascular anatomy and the persistent risk of rupture, the interventional radiology team reviewed the case. Two options were considered: repeat endovascular embolization at a tertiary center or local percutaneous thrombin injection under imaging guidance. After discussion of the respective risks and benefits, the patient elected to proceed with the locally available percutaneous thrombin injection.

The following day, CT-guided thrombin injection of the pseudoaneurysm was performed under general anesthesia. Preprocedural coagulation studies confirmed an international normalized ratio of 1.2 and a normal platelet count, supporting the safety of proceeding with the intervention. A 20-cm, 20-gauge Chiba needle was advanced percutaneously into the pseudoaneurysm sac under CT guidance, and 1000 IU of human thrombin was injected. Initial low resistance to injection followed by increasing resistance suggested successful intraluminal thrombus formation (Figure 2). Although postprocedural arterial-phase imaging was not obtained because of technical limitations and contrast load, delayed-phase imaging demonstrated no significant opacification of the pseudoaneurysm sac, consistent with successful embolization.

**Figure 2:**
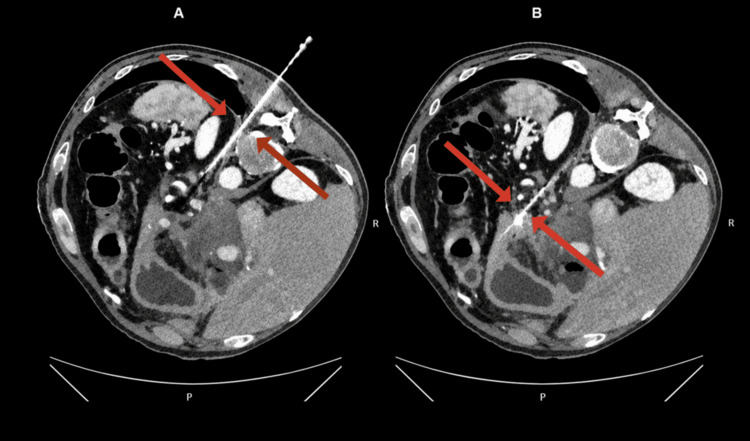
Axial CT obtained during the CT-guided procedure showing the 20-cm, 20-gauge Chiba needle positioned within the pseudoaneurysm sac before thrombin injection

The day after the thrombin injection, the patient reported marked improvement in abdominal pain. He remained hemodynamically stable, with improving liver function tests and inflammatory markers, and stable hemoglobin without clinical features of hemorrhage. He was therefore considered suitable for transfer back to his regional hospital for ongoing monitoring and planned interval imaging to confirm persistent pseudoaneurysm occlusion.

Follow-up arterial-phase CT angiography five days later demonstrated that the previously identified pseudoaneurysm in the left gastric artery territory no longer opacified. In its place, a 7.5-cm pancreatic pseudocyst was identified at the porta hepatis, interposed between the caudate lobe, inferior vena cava, and aorta, causing mass effect on the portal confluence but without thrombosis (Figure 3). Given the absence of thrombosis or other high-risk features, an initial conservative approach with observation was adopted. However, in view of the pseudocyst’s size and the patient’s ongoing discomfort, he was subsequently scheduled for image-guided drainage two weeks after its identification.

**Figure 3:**
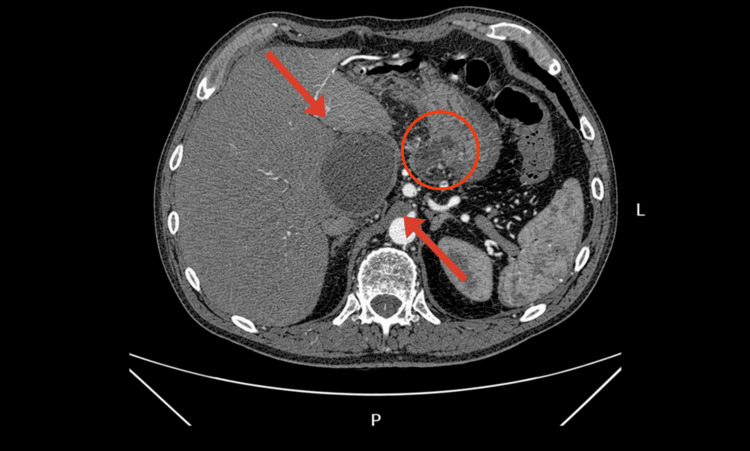
Arterial-phase CT angiography demonstrating nonopacification of the previously identified pseudoaneurysm, now thrombosed (circled), in the left gastric artery territory and a 7.5-cm pancreatic pseudocyst (arrow)

Under ultrasound guidance and local anesthesia, a 19-gauge needle was advanced transhepatically through the left hepatic lobe into the pancreatic pseudocyst, and an 8.5-French pigtail catheter was inserted using a standard Seldinger technique and secured in situ. The drain functioned well.

The following day, the patient was discharged home with community nursing support for regular catheter flushing and scheduled follow-up imaging. One month later, CT of the abdomen and pelvis showed interval clearance of the pancreatic pseudocyst, with the transhepatic pigtail catheter still in situ, no recurrence of the pseudoaneurysm, and no new collections or features of active pancreatitis (Figure 4). The drain was subsequently removed. Additionally, at this one-month follow-up, liver function tests had normalized, and hemoglobin remained stable (approximately 119 g/L), with no recurrence of the pseudoaneurysm or new collections.

**Figure 4:**
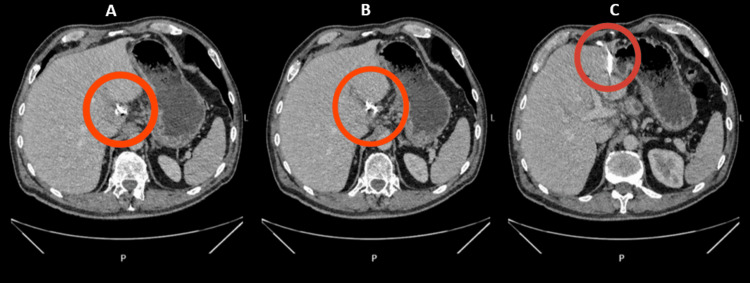
Portal venous-phase contrast-enhanced CT of the abdomen and pelvis showing interval clearance (A-C) of the pancreatic pseudocyst with the transhepatic pigtail catheter in situ

Follow-up CT imaging four weeks later demonstrated interval removal of the transhepatic drain, no residual fluid collection, and no opacification of the previously treated pseudoaneurysm. There was no CT evidence of active pancreatitis, indicating radiologic and clinical treatment success (Figure 5).

**Figure 5:**
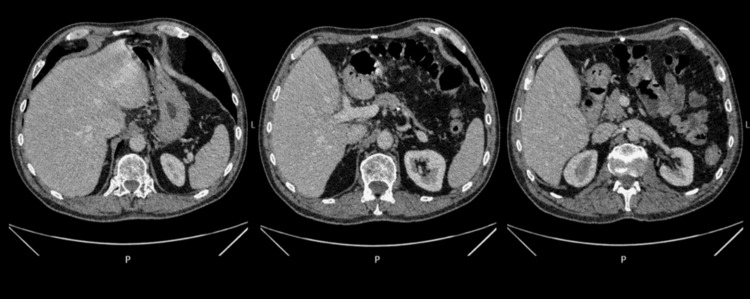
Portal venous-phase contrast-enhanced CT of the abdomen and pelvis showing no residual fluid collection, no opacification of the prior pseudoaneurysm, and no evidence of active pancreatitis

A summary of the key clinical events and imaging studies is provided in Table 1.

**Table 1:** Timeline of key clinical events DSA, digital subtraction angiography

Day from presentation	Event
Day 0	Presentation to the regional hospital with epigastric pain and melena; hemodynamically stable
Day 2	CT of the abdomen showing acute pancreatitis with a suspected visceral pseudoaneurysm
Day 5	Transfer to a higher-level center for consideration of embolization
Day 7	DSA confirmed a left gastric artery pseudoaneurysm; attempted endovascular embolization was unsuccessful
Day 8	CT-guided thrombin injection performed under general anesthesia
Day 9	Clinical improvement; hemodynamically stable; repatriated to the regional hospital
Day 14	Follow-up CT angiography: pseudoaneurysm no longer opacified; large porta hepatis pancreatic pseudocyst identified
Day 35	Planned admission for ultrasound-guided transhepatic drainage of the porta hepatis pancreatic pseudocyst with pigtail catheter insertion
Day 49	CT of the abdomen and pelvis: interval evacuation of the pancreatic pseudocyst with the transhepatic pigtail catheter in situ
Day 83	CT of the abdomen and pelvis: interval removal of the transhepatic drain; no residual collection and no opacification of the treated pseudoaneurysm

## Discussion

This case highlights a rare but clinically significant complication of pancreatitis. The patient presented with epigastric pain and GI bleeding, and imaging identified a visceral artery pseudoaneurysm arising in the setting of acute pancreatitis. Visceral artery pseudoaneurysms are uncommon but potentially life-threatening sequelae of pancreatitis, with a high risk of rupture if not recognized and treated promptly. Although DSA remains the gold standard for diagnosis and often enables therapeutic embolization [1,3], catheterization of the feeding artery in this case was unsuccessful because of vessel tortuosity, rendering standard endovascular management unfeasible.

CT-guided thrombin injection was therefore selected as a minimally invasive alternative. Originally described for the treatment of iatrogenic femoral pseudoaneurysms, image-guided thrombin injection has increasingly been adopted in visceral applications when anatomic constraints preclude conventional transarterial embolization [7,8]. Published reports of left gastric and peripancreatic pseudoaneurysms treated with thrombin injection describe high technical and clinical success rates with relatively low complication rates in experienced hands. This case adds to the growing body of evidence supporting CT-guided thrombin injection as a feasible minimally invasive option in selected cases where standard endovascular embolization is not possible [4,8,9].

Despite its advantages, thrombin injection carries recognized risks, including nontarget embolization, distal arterial thrombosis, and pseudoaneurysm recurrence [6,7]. Careful preprocedural planning, meticulous needle positioning, and real-time imaging guidance are therefore essential to minimize complications. In addition, postprocedural cross-sectional imaging is crucial both to confirm complete thrombosis of the pseudoaneurysm sac and to detect early recurrence or treatment-related sequelae. In this patient, follow-up CT angiography demonstrated sustained exclusion of the pseudoaneurysm, with immediate occlusion inferred from delayed-phase rather than arterial-phase imaging at the index procedure (arterial-phase confirmation was not obtained because of technical issues), no opacification on serial studies, and no clinical features of rebleeding over approximately two months of imaging follow-up. Structured surveillance also revealed a large porta hepatis pancreatic pseudocyst that required subsequent image-guided drainage. Nevertheless, interpretation of this outcome is limited by the single-patient nature of the report and the absence of immediate arterial-phase imaging following thrombin injection, which precluded direct confirmation of instantaneous sac thrombosis. Together, these findings emphasize that posttreatment surveillance imaging is important not only to confirm pseudoaneurysm occlusion but also to enable early detection and management of postprocedural complications, such as the pancreatic pseudocyst observed in this case.

## Conclusions

Overall, this case suggests that CT-guided percutaneous thrombin injection may be a useful adjunct in the management of visceral pseudoaneurysms when conventional endovascular techniques are not viable. It reinforces the importance of a multidisciplinary approach integrating interventional radiology and surgical teams and illustrates that, with careful patient selection and structured imaging follow-up, minimally invasive techniques can achieve favorable outcomes in complex pancreatitis-related vascular complications. However, these conclusions are drawn from a single case and must be interpreted in light of the limited follow-up.
